# Observations on the Tread-Wheel

**Published:** 1823-09

**Authors:** J. M. Good


Art. VIII.—*Observations on the Tread-Wheel.* Contained in a Letter to the Editors, by J. M. Good, M.D. F.R.S. &c. &c.

I am glad to find, by an introduction of Mr. Hutchinson's letter to you on the discipline of the tread-wheel, that you feel this subject of sufficient importance, in a medical point of view, to be brought before the public. The short address from Sir John Cox Hippisley, Bart. to his friend the Minister of the Home Department, upon which this letter is founded, and in the drawing up of which I was called upon professionally to take a part, was written with considerable haste, and when the subject was new, in order that its contents might be laid before the assembled magistrates of most of the counties of England, at the last Epiphany sessions; and for this purpose it was put gratuitously into circulation, but never published. Since this period, so much additional matter of considerable importance, and fully establishing the facts and opinions of the unpublished address, have been obtained by ~~the same distinguished statesman~~ that he has felt it his duty to put the whole together in a second address to Mr. Peel, and to lay it before the public. I herewith beg your acceptance of a copy of this address, and shall take care to forward another copy of it to Mr. Hutchinson, in order that he may be put into possession of the whole case; and shall leave it to your own judgment to bring the entire question before your readers, in whatever way you may think best adapted for the purpose of free discussion; and, so long as such discussion is conducted in the spirit of candour and liberality which Mr. Hutchinson has evinced, it cannot fail of being highly instrumental to the improvement of prison discipline, and of developing many points of importance within the range of forensic medicine.

How long the tread-wheel has been established in the House of Correction at Southwell, or how many are, upon an average, employed upon it, though points of the utmost moment in the present inquiry, are not even glanced at by Mr. Hutchinson in his letter upon the subject; who seems also to have forgotten to mention whether females are sentenced to the same labour or not. As no communication from the visiting justices of the Nottinghamshire House of Correction appears among the official returns, by order of the House of Commons, made to the Secretary of State for the Home Department, respecting the use of tread-wheels in all gaols or houses of correction, in which they were established on January 18th of the present year, it is most probable that this establishment has taken place *since;* and, as one of the chief difficulties we have had to encounter is the very short period of time in which this instrument has been at work *any where*, it is obvious that no general conclusion could be drawn from what has occurred in the house of correction before us, had the scale of employment been even much larger than there is reason to calculate it at, and had female prisoners been subjected to the labour; which it does not appear that Mr. Hutchinson has ventured to recommend, notwithstanding his general approbation of the tread-wheel discipline.

Beyond the walls of this prison, and the period of time in which the tread-machine has been working there, Mr. Hutchinson's experience does not seem to have travelled; and hence, so far as relates to the *facts* he has been an eye-witness to, they form no collision whatever with the mass of *general facts* and *opinions* brought forward in Sir John Hippisley's publication, which makes ample allowance for exemptions and procrastinated evils; while the *tendency* to mischief still continues to operate, and has sufficiently shown itself wherever there has been time and opportunity.

How far Mr. Hutchinson's views of the effects of such a kind of exercise as the tread-wheel imposes, in relation to hernias and varicose tumors of the legs, may be correct, he will learn, and the public also will learn, from an extensive and interesting branch of inquiry, contained in Sir John's pamphlet, directly bearing upon this subject, and probably new to many of your readers. Mr. Hutchinson, however, is quite correct in stating that the prisoners at work upon the wheel have it in their power, instead of treading with their toes, or the fore-part of the foot only, to twist their knees outward, and bring a larger portion of the foot into action, without which he is ready to admit that the exercise "could be continued but a very short time, and would be productive of never-ending lameness and misery to the prisoner who had suffered this torture." In the pamphlet now sent you will find, however, that, though this change of position can be accomplished, and is accomplished, for a few moments at times, it is not persevered in by the prisoners in Cold-bath Fields, who are as dextrous as most in the kingdom, and cannot be persevered in for more than a few moments at a time, on account of the pain which such a twist of the knees produces, and which compels the prisoner to return abruptly to his original and ordinary bearing upon the fore-part of the foot alone: and hence, indeed, the violent heat, exhaustion, and perspiration, into which he is constantly thrown by an exercise of not more than ten minutes or a quarter of an hour, although his progression is so slow, that his entire day's walk up hill, and this without any burden to carry, does not exceed *two miles* or *two miles and a half for the whole day*: and hence also that necessity which is found every where, on the introduction of tread-wheels, for a richer diet. This at present consists, in the House of Correction in Cold-bath Fields, of half a pound of solid flesh every other day, with good animal soup in the intermediate day, besides a sufficiency of bread and other farinaceous food; and Mr. Webb, the prison-surgeon, whose official Report shows ostensibly that he is not unfriendly to the tread-wheel, has told me, within a very few days, that, without this increased diet, the workers on the wheel would be soon in the situation of the convicts at the Millbank Penitentiary. All which facts speak sufficiently for themselves.

It was in order to determine how far it might be adviseable to enforce the erection of tread-wheels in all our prisons by a parliamentary enactment, that the visiting magistrates were lately called upon, by the order of the House of Commons just adverted to, to make returns to the office of the Home Secretary of the effects actually produced, wherever they had obtained an establishment. Mr. Hutchinson conceives that "this mass of most respectable evidence speaks loudly in favour of the highly salutary and safe operation of this mode of preventing a repetition of crime." He will find, in the pamphlet I am about to send him, that there are others who have examined it very accurately, and think differently; and, by turning to the third volume, p. 151, of the "Medical Jurisprudence" of Dr. Paris and Mr. Fonblanque, he will also find that the individuals whose opinions are given in Sir John Hippisley's pamphlet, are not the only ones who think differently; for the same general views of the mischiefs of the tread-wheel discipline are taken by those able and intelligent writers with the official reports before the public. But, which is of far more weight upon the subject, by turning to the newly-passed Gaol Act, the passage of which through the legislature was delayed till these returns were received, in order that it might contain certain clauses enforcing or recommending an adoption of the tread-wheel generally, if they should be found to justify such a measure: he will perceive that, so differently have his Majesty's ministers, and even Parliament itself, thought of the result of these communications, that every such clause was withdrawn from the first; the treadwheel has been abandoned by the statute; and not one single word urged in its favour during the long passing of the Bill backwards and forwards through the two houses. Nor can any thing exceed the candour which has been exhibited by his Majesty's ministers upon this point, because it is very well known that several of them were antecedently most strenuous advocates for the tread-wheel machine.*

*Guildford-street; August* 4, 1823.

### COLLECTANEA MEDICA:

* It is our intention, in the ensuing Number, to present our readers with an account of Sir J. HIPPESLEY's work, alluded to in the above letter.—EDITORS.

