# Erratum in the Last Number

**Published:** 1823-10

**Authors:** 


Erratum *in the last Number.*—In Dr. Good's Communication, line 15, for "*the same distinguished statesman,*" read "Sir J. C, Hippisley."

